# Electroacupuncture Improves Antidepressant Effects in CUMS Rats by Protecting Hippocampal Synapse and Mitochondrion: An Ultrastructural and iTRAQ Proteomic Study

**DOI:** 10.1155/2019/3424698

**Published:** 2019-04-17

**Authors:** Jialing Zhang, Zhinan Zhang, Jiping Zhang, Zheng Zhong, Zengyu Yao, Shanshan Qu, Yong Huang

**Affiliations:** ^1^School of Chinese Medicine, The University of Hong Kong, 999077, Hong Kong; ^2^School of Traditional Chinese Medicine, Southern Medical University, Guangzhou, Guangdong Province 510515, China; ^3^Nanfang Hospital, Southern Medical University, Guangzhou, Guangdong Province 510515, China

## Abstract

Electroacupuncture (EA) is considered a complementary therapy for depression. Trials also found that EA has additive benefits when combined with medication compared with medication alone. It is revealed that EA restores altered hippocampal synaptic plasticity in depressed brain. But precise molecular mechanism is poorly understood. Here, we evaluated the therapeutic effects of EA and EA combined with selective serotonin reuptake inhibitor (SSRI) on depressed (CUMS) rats. Then a new proteomics approach, isobaric tag for relative and absolute quantitation (iTRAQ), was used to explore the differential expressed synaptic protein in hippocampus between CUMS and EA-treated rats to identify the possible target molecular mechanism of its effects. We found that EA had additive benefit against depressive behaviors when combined with SSRI. Ultrastructure study on neuron showed significant change in postsynapse density (PSD) and mitochondrion. Through iTRAQ, it is found that synaptic and mitochondrial proteins were significantly changed after EA, consisting with ultrastructure study results. These findings suggest that EA improves antidepressant performance in depressed rats through protecting synaptic and mitochondrial functions in hippocampus.

## 1. Introduction

Although antidepressants instantly increase levels of monoamines between synapse clefts, it takes weeks until symptoms alleviation [1, 2]. Besides, the efficacy of antidepressants may be outweighed by their adverse health effects [3]. Therefore, therapies that overcome these limits are rather expected.

Electroacupuncture (EA) is a form of acupuncture where a small electric current is passed between pairs of acupuncture needles [4, 5]. Findings suggest that EA combined with antidepressant brings the symptomatic improvement of antidepressants treatment forward within 1-2 weeks with less adverse health effects of antidepressant [6, 7]. Moreover, our previous RCT study shows that combining EA (at GV20 and GV29) with paroxetine (a widely used SSRI) improves depressive symptoms within at least 1 week and the therapeutic effects continue even after the therapy [8, 9]. Therefore, combination therapy of EA and antidepressants is a promising strategy against depression.

Hippocampal volume loss is one of the neuroanatomical characters of depression [10, 11]. Thus, hippocampus is considered as a diagnostic neurobiomarker and a therapeutic target for depression [12]. MRI researches also prove that hippocampus is one of the target brain regions of EA in depressed patients [13–15].

A prominent feature of hippocampus is synaptic plasticity [16, 17]. Recent studies have called attention to the role of altered hippocampal synaptic plasticity in the biology of depression. It is showed that chronic stress reduces synaptic and dendritic plasticity. Moreover, depressed subjects show evidence of impaired neuroplasticity and antidepressant medications enhance neuroplasticity at both a molecular and a dendritic level. These findings suggest that disrupted synaptic plasticity is an underlying feature of depression [18, 19]. Besides, mitochondria are enriched at synapse as supporting organelles. It is also revealed that mitochondria play an active role in synaptic plasticity through multiple pathways [20]. Researches show that EA relieves depression by restoring hippocampus synaptic plasticity in hippocampus through increasing glutamate receptor or decreased serotonin receptor, proposing the importance of hippocampal synaptic plasticity in the acting mechanism of EA [21–23]. Researches also show that EA intervention can reverse mitochondria damage in depressed hippocampus [24]. However, there exact molecular mechanism remains largely to be discussed. We therefore sought to thoroughly investigate synaptic as well as mitochondrial proteins involved in the synaptic regulation of EA on depressed hippocampus.

Proteomic technologies are the ideal techniques for the detection and investigation of biomarker candidates, owing to the high sensitivity and analytical performance that can be achieved and the ability to generate large datasets through identification of large and ever-increasing numbers of proteins [25–27]. Isobaric tag for relative and absolute quantitation (iTRAQ) is a proteomics approach which can determine the amount of proteins from different sources in a single experiment [28]. This technology has been used to outline the proteomics profiles of cancers [29]. Currently, a number of biomarkers have been detected in urine and tissue for bladder cancer using this technique [30]. Therefore, we used iTRAQ to thoroughly explore the mechanism of EA's effects on hippocampal synaptic plasticity in depression.

In this study, we perform EA at GV20 (Baihui) and GV29 (Yintang) in depressed (CUMS) rats, using behavioral tests to monitor the effects of EA on depressive-like symptoms. We also observed the synaptic ultrastructure differences between CUMS and EA hippocampal neurons. iTRAQ was used to identify the differentially expressed hippocampal synaptic proteins between CUMS and EA rats to identify candidate proteins responsible for the therapeutic effects of EA on depression.

## 2. Material and Methods

### 2.1. Animals

70 male Wistar rats (weight 200±20g, 6-7-week-old, Experimental Animal Center of Southern Medical University) were housed in individual cages under a 12-12 h light/dark cycle in a standard SPF facility (temperature 24±2°C; humidity 50%-60%) with access to food and water ad libitum. Before we started the experiment, rats were allowed to habituate for 6 days. Rats were randomly assigned into 5 groups: control, CUMS, SSRI, EA, and EA+SSRI groups (n=10-15 rats/group). All the experimental procedures were performed in accordance with the National Institutes of Health Guide for the Care and Use of Laboratory Animals and the procedures were approved by the Ethics Committee on Animal Experimentation of Southern Medical University. All efforts were made to minimize animal suffering and to reduce the number of animals used.

### 2.2. CUMS Paradigm

**Figure 1:**
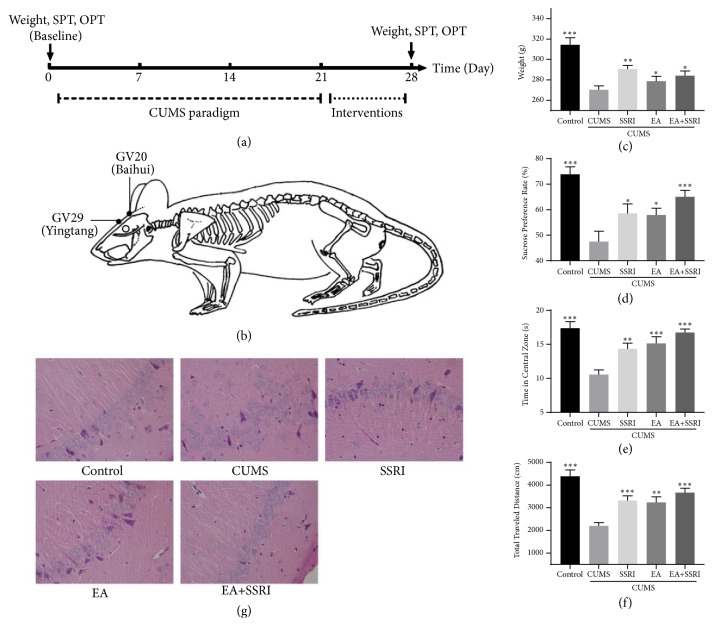
EA+SSRI accelerates depressed-like behaviors improvement in depressed rats. (a) Timeline of CUMS paradigm, interventions, and behavioral tests. (b) EA was performed at acupuncture point GV20 (Baihui) and GV29 (Yingtang). (c) EA and EA+SSRI increased body weight change caused by CUMS. (d) Both EA and EA+SSRI increased sucrose preference rate in depressed rats. EA+SSRI increased sucrose preference significantly. (e) EA and EA+SSRI increased central zone time spent in depressed rats significantly. (f) CUMS-induced total traveled distance decrease in open field was reversed by EA and EA+SSRI. EA+SSRI increased total traveled distance significantly (n=10-15 rats/group). (g) EA+SSRI reverses the hippocampal histopathology changes during CUMS (n=6 rats/group), × 400. Bar graphs represent mean ± SE. *∗*: P<0.05, *∗∗*: P<0.01, and *∗∗∗*: P<0.001 vs. CUMS. One-way ANOVA with LSD post hoc test.

To develop a CUMS rat model, rats were exposed to 10 different kinds of stressors over a period of 21 days, 1 stressor per day (Figure 1(a)). All of these stressors were random arranged, which include water deprivation (24 h), food deprivation (24 h), wet bedding (24 h), light-dark reversal (24 h), Stroboscopic lighting (12h), immobilization (2 h), cold swim (4°C, 5min), warm swim (45°C, 5min), level shaking (5min), and tail clamping (3min) [31, 32].

### 2.3. Interventions

After CUMS paradigm, rats in EA group underwent EA at GV20 (above the apex auriculate, on the midline of the head) and GV29 (at the middle point between the eyes) (Figure 1(b)) [33]. Rats were lightly immobilized with plastic fixator. Then disposable acupuncture needles (0.30mm × 25mm, Hua Tuo Appliance Factory) were inserted in both acupoints obliquely to 5mm depth. Following the insertions, electrodes were added to the handle of the needles using 1mA, 2Hz, and 5Hz, electrical simulation for 30 mins each time, once per day for 7 consecutive days. EA rats were also given saline (same volume per kg as paroxetine). Rats in SSRI group were given paroxetine (1.8mg/kg/d) [34, 35] after 30-minute immobilization for 7 consecutive days. Rats in EA+SSRI group were given both EA intervention and paroxetine once per day for 7 consecutive days. For the CUMS group and control group, we only performed lightly immobilization and saline administration, once per day during the same period.

### 2.4. Behavioral Assessments

In sucrose preference test (SPT), rats were subjected to a 2-day habituation phase before the actual test phase. In the 3^rd^ day, food and water were removed for a deprivation period of 23 h. The following morning, test phase began, during which customized drinking bottles filled with either 1 % sucrose solution or water were given to the rats for 1 h. Sucrose preference rate was calculated as the amount of sucrose solution relative to the total fluid consumption [36].

In open field test (OFT), we used an arena with walls in a quiet and dimly lighted room as the open field (OF). The field was divided into 25 equally with grids and square crossings. Center zone dominated the 9 grids in center, and other 16 grids belonged to peripheral zone. Each rat was put in the center of the OF for a period a 5mins, during which the tester left the room. We used video cameras with associated software (Smart 2.0) to automate the assessment process. Behavioral patterns measured include time in central zone and total travel distance [37].

Behavioral tests and weight assessment were performed after intervention termination.

### 2.5. Tissue Processing

After animals were anesthetized with 25% pentobarbital sodium (50mg/kg, intraperitoneal injection), we used cervical dislocation to prevent pre- and postsynaptic effects of anesthesia. Animals were decapitated and the brains were instantly dissected with all attached tissues removed. Hippocampus tissues were removed and rinsed with pre-cold phosphate-buffered saline (PBS). Removed tissues of 3 rats/group were immediately snap-frozen within liquid nitrogen and stored in refrigerator (-80°C) until isobaric labeling. 6 rats/group were transferred into in the fixative (4% paraformaldehyde; 0.1 M phosphate buffer at pH 7.4) until electron microscopy and HE stain.

### 2.6. Electron Microscopy

Tissues were microwaved at full power (700W microwave oven) for 10 s to enhance penetration of the fixative through the depth of the slice with a final temperature of <35°C. Slices were left overnight in the same fixative and then rinsed with 0.1M phosphate buffer (4× for 15 min). First, they were immersed in 1% osmium for 1 hour and rinsed in buffer (3× for 15 min). Next, they were immersed in ascending concentrations of acetone (50, 70, 90, and 100%). Finally, they were quickly immersed in Spurr's resin at room temperature overnight and then embedded in coffin molds in Spurr's resin and cured for 8 hours at 70°C in an oven. They were then vibrasliced at 60 nm (Leica UC7, Leica). Ultrathin sections were counterstained with saturated aqueous uranyl acetate, followed by Reynolds lead citrate for 5 min. Sections were photographed with a transmission electron radiography (Hitachi H-7500, Hitachi) at 5,000× magnification.

### 2.7. Sample Preparation and Isobaric Labeling

Equal amounts of each hippocampus sample were pooled to produce a sample group. Samples were randomly mixed into a sample group as a biological replicate. The hippocampus samples were reduced and then alkylated, followed by determination of protein concentration in the samples using bicinchoninic acid assay (H4MFPTAD, BioTek). 100 mg protein from each sample was digested with trypsin (Promega) overnight at 37°C. The pH was monitored to assure a complete digestion. After trypsin digestion, different sample peptides resolved in 0.5 M TEAB (Sigma) were labeled with different isobaric tags according to the protocol of the iTRAQ Reagent-8plex Multiplex Kit (AB SCIEX). Briefly, one unit of iTRAQ reagent was thawed and reconstituted in 50 *μ*L isopropanol. Peptides from groups were labeled with different iTRAQ tags by incubating at room temperature for 2 h. The peptide mixtures were then pooled and dried by vacuum centrifugation.

### 2.8. Fractionation by SCX Chromatography

For SCX chromatography, we used the HPLC (L-3120, Rigol). The peptides were eluted at a flow rate of 700 *μ*L/min with a gradient of buffer A (0.1% methane acid, Sigma) and then buffer B (0.1% methane acid; 80% acetonitrile, Sigma). Peptides were equilibrated with buffer A prior to the next injection. Elution was monitored by measuring absorbance at 5 min, and fractions were collected every 1.5 min. The eluted peptides were pooled as 40 fractions, freeze-centrifuged, and vacuum-dried. Labeled samples were then pooled and dried in a vacuum concentrator. The pooled mixtures of iTRAQ-labeled peptides were fractionated by strong cation-exchange chromatography (L-3120, Rigol) for LC-MS/MS.

### 2.9. LC-ESI-MS/MS Analysis

Each fraction was resuspended in a volume of 5ul 0.5% FA (Sigma) and centrifuged at 14,000 g for 10 min. In each fraction, the final concentration of peptide was approximately 0.5 mg/mL on average. In total, 10 *μ*L of each supernatant was loaded on a HPLC Ultimate 3000 (Thermo Scientific) with an autosampler onto a C18 trap column (C18 3*μ*m 0.10×20mm), and the peptides were eluted onto an analytical C18 column (C18 1.9*μ*m 0.15×120mm) packed in-house. The samples were loaded at 600nl/min. Data acquisition was performed with a Q-Exactive HF (Thermo Finnigan).

### 2.10. iTRAQ Protein Identification and Quantification

The original MS/MS file data (*∗*.RAW) were analyzed with Mascot 2.1 and Proteome Discoverer 1.4 (Thermo) using the Rat database download from Uniprot database on Apr. 15th, 2016. The search parameters included the following: Enzyme = Trypsin; Max Missed Cleavages = 2; Fixed modifications = Carbamidomethyl (C); Variable modifications = Oxidation (M), Acetyl (Protein N-term), iTRAQ8plex (N-term), iTRAQ8plex (K), and iTRAQ8plex (Y); Peptide Mass Tolerance = ± 15 ppm; Fragment Mass Tolerance = 20mmu; peptide confidence = high. Peptide FDR≤0.01 was used as the identification standard. A 1.2-fold cutoff was set to identify upregulated and downregulated proteins.

### 2.11. Bioinformatics Analysis

The proteins were analyzed using on-line analysis tools. Gene ontology (GO) and KEGG pathway analyses were performed through the STRING website (http://string-db.org/), and separate figures were produced for biological process, molecular function, and cellular component categories. The differentially expressed protein-protein network was analyzed by STRING website (http://string-db.org/) and the figure was also produced by the software.

### 2.12. Statistical Analysis

All data are expressed as the mean ± SEM. Differences in body weight change, sucrose preference rate, time in center zone, and total travel distance between groups (control, CUMS, and EA groups) were evaluated for statistically significant differences using One-way analysis of variance (ANOVA) followed by Least-Significant Difference (LSD) post hoc tests, or Dunnett-T post hoc test, if there was heteroscedasticity presence (SPSS software, Version20.0, SPSS Inc., USA). P < 0.05 was considered statistically significant.

## 3. Results

### 3.1. EA Accelerates SSRI's Antidepressant Effects in Depressed Rats

After a 21-day chronic unpredictable mild stress (CUMS) paradigm, rats underwent EA at GV20 and GV29 or EA+SSRI for 7 days (Figures 1(a)-1(b)). And we used behavioral tests to assess therapeutic effects. Body weight, sucrose preference test (SPT), and open field test (OFT) were used to assay appetite, anhedonia, and anxiety change, respectively.

After 7-day interventions, we found that EA alone has similar effects as SSRI in decreasing anhedonia and anxiety (Figures 1(c)-1(f)). In body weight changing, SSRI performed better than EA and EA+SSRI (Figure 1(c)). Additionally, EA had additive benefits when combined with SSRI compared with medication alone in decreasing anhedonia and anxiety, which consist with our previous trail results. For instance, compared with group EA or SSRI, weight, sucrose preference rate, travel distance, and central zone time spent of group EA+SSRI were significantly increased (Figures 1(d)-1(f)). Thus, these results suggest that combination of EA with SSRI improves anhedonia and anxiety in depression better than EA or SSRI alone. SSRI alone increases weight better than EA or EA+SSRI.

To further ascertain the efficacy of the EA and EA+SSRI, the histopathology of hippocampus was evaluated with HE stains. In control group, the hippocampal pyramidal cell layer in cornu Ammonis (CA) 1 is thick; cells are densely, closely, and regularly arranged; the cellular structure is complete with a clear edge. In CUMS group, the hippocampal pyramidal cell layer is thin; intercellular spaces are widened; cells are irregularly and loosely arranged; the cellular structure is incomplete, even with loss of large amounts of cells, indicating that the hippocampal tissue was damaged and cell apoptosis occurred in CUMS group. In SSRI, EA, and EA+SSRI groups, the hippocampal pyramidal cell layer is restored and intercellular spaces are narrowed to some extent, indicating rehabilitative effect against damage in the hippocampus (Figure 1(g)).

### 3.2. EA+SSRI Reverses Neuron Ultrastructure Pathology in Hippocampus of Depressed Rats

**Figure 2:**
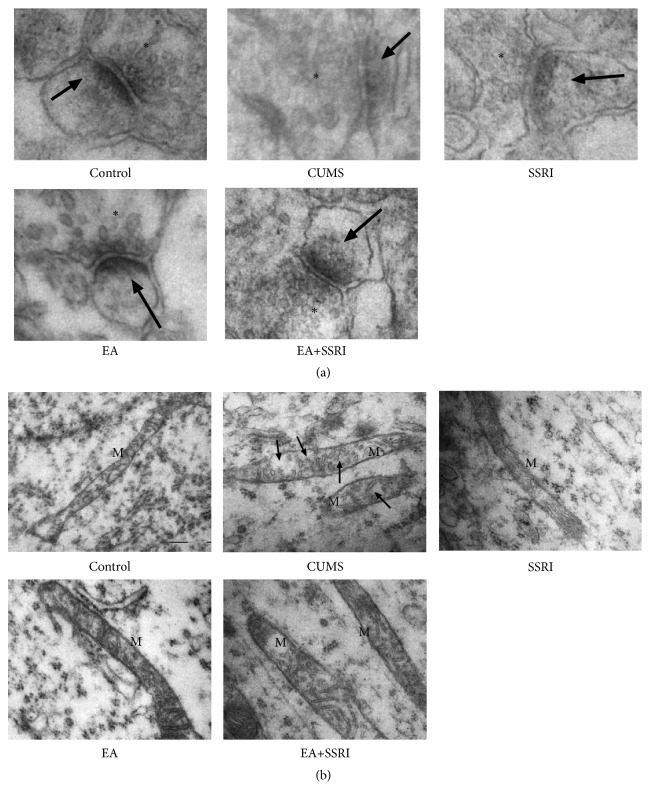
EA+SSRI reverses postsynaptic density loss and mitochondrial lesion during depression. (a) CUMS caused PSD loss in hippocampal neuron. EA and SSRI alone reversed the loss while EA was more effective. EA+SSRI induced more PSD increase compared with EA or SSRI alone. Asterisks (*∗*): presynaptic vesicles; arrowheads: postsynaptic density (n = 3 rats/group); × 80,000. (b) Exposed to CUMS, mitochondria in hippocampal neuron were enlarged and contain fragments of cristae. EA, SSRI, and EA+SSRI reversed mitochondrial damage (n = 3 rats/group). M: mitochondria. Arrowheads: cristae dissolve; × 24,000.

Increasing evidence shows that the biological mechanism of depression lies in synaptic plasticity, especially in prefrontal cortex and hippocampus [18, 19]. To investigate the role of EA in CUMS-induced synaptic plasticity change, we examined the ultrastructure of hippocampal synapse through transmission electron microscopy. Significant loss of PSD was observed in CUMS rats. Both EA and SSRI alone reversed the loss while EA caused a more significant increase in PSD. When combined with SSRI, EA caused more PSD increase compared with EA or SSRI alone (Figure 2(a)). These findings suggest that synaptic plasticity especially postsynaptic structure is changed during interventions. EA, SSRI, and combination of EA with SSRI reverse PSD loss caused by CUMS, and combination treatment has the best effects.

We also found that, exposed to CUMS, mitochondrial ultrastructures in hippocampal neuron were also damaged. Normally, mitochondria are rod shaped organelles with double membrane bound (the outer membrane and the inner membrane). CUMS caused mitochondria to enlarge and cristae dissolve and disappear leaving empty intermembrane space. EA, SSRI, and combination of them provoked mitochondria repair (Figure 2(b)). These findings indicate that mitochondria repair is also involved in the regulation of EA against depression.

### 3.3. EA Changes Hippocampus Proteomics Profiles of Depressive Rats

To investigate the molecular mechanisms underlying the effects of EA in depression, we examined the differentially expressed hippocampal proteins between control, CUMS, and EA groups through iTRAQ and used bioinformatics analysis methods to understand proteomics characters variations.

The protein abundance of 274 proteins (145 upregulated and 129 downregulated) identified with iTRAQ-based technology showed greater than a 1.2-fold change or less than a 0.83-fold change when comparing EA to CUMS rats, as well as 52 proteins (52 upregulated and 22 downregulated) when comparing CUMS to control (Table 1).

#### 3.3.1. Categorization of Differential Proteins

**Figure 3:**
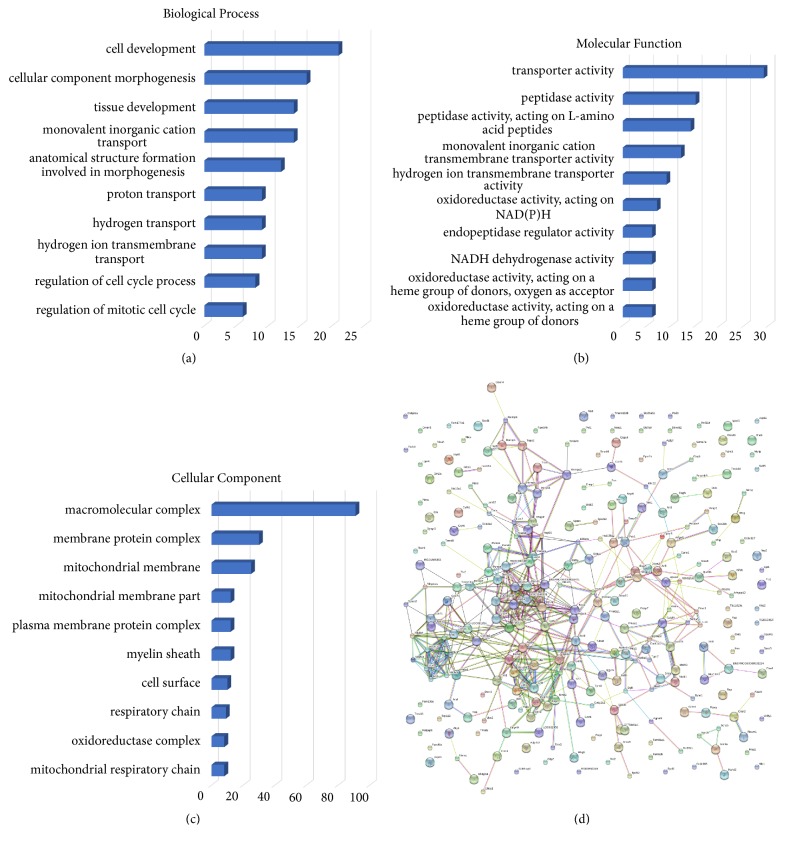
EA changes hippocampus proteomics characters in depressed rats. (a)-(c) Bar charts of the GO annotation of different proteins comparing groups. Figures (a), (b), and (c) denote biological process, cellular component, and molecular function of differential expressed proteins between CUMS and control rats' hippocampus, respectively. Scale bar: number of proteins. (b) Protein-protein interactions identified comparing EA and CUMS groups. Network of EA/CUMS has significantly more interactions than expected in STRING analysis. Network nodes: proteins; edges: associations (stronger associations are represented by thicker lines).

To understand variations in the proteomic characteristics of differentially expressed hippocampal proteins between CUMS and EA, differentially expressed proteins were subjected to GO analysis in the STRING website (version 10). The results show that, in terms of biological process, most of the differential expressed proteins were mainly involved in cell development (8.30%, P=0.03), cellular component morphogenesis (6.32%, P=0.04), tissue development (5.53%, P=0.01), monovalent inorganic cation transport (5.53%, P=0.01), anatomical structure formation involved in morphogenesis (4.74%, P=0.01), proton transport (3.55%, P=0.01), hydrogen transport (3.55%, P=0.01), hydrogen ion transmembrane transport (3.55%, P=0.01), regulation of cell cycle process (3.16%, P=0.01), and regulation of mitotic cell cycle (2.371542%, P=0.04) (Figure 3(a)). Regarding molecular function, most of the differential proteins were annotated as being associated with transporter activity (11.46%, P=0.04), peptidase activity (5.92%, P=0.03), peptidase activity, acting on L-amino acid peptides (5.53%, P=0.04), monovalent inorganic cation transmembrane transporter activity (4.74%, P=0.03), hydrogen ion transmembrane transporter activity (3.55%, P<0.01), oxidoreductase activity, acting on NAD(P)H (2.76%, P=0.02), endopeptidase regulator activity (2.37%, P=0.03), NADH dehydrogenase activity (2.37%, P=0.01), oxidoreductase activity, acting on a heme group of donors, oxygen as acceptor (2.37%, P<0.001), oxidoreductase activity, and acting on a heme group of donors (2.37%, P<0.001) (Figure 3(b)). In terms of cellular component, most of the differential proteins are predicted to be in macromolecular complex (35.96%, P=0.03), membrane protein complex (11.85%, P=0.045781), mitochondrial membrane (9.88%, P=0.04), mitochondrial membrane part (4.74%, P=0.04), plasma membrane protein complex (4.74%, P=0.03), myelin sheath (4.74%, P=0.03), cell surface (3.95%, P=0.02), respiratory chain (3.55%, P=0.01), oxidoreductase complex (3.16%, P=0.04), and mitochondrial respiratory chain (3.16%, P=0.01) (Figure 3(c)).

#### 3.3.2. Pathways Relevant to Differential Hippocampal Proteins

To explore the connection of differential proteins between CUMS and EA hippocampus, KEGG pathway mapping was analyzed. KEGG pathway mapping revealed 5 significant pathways involved in EA-CUMS comparison (Table 2). The pathways were nonalcoholic fatty liver disease (NAFLD) (12 proteins, P<0.001), oxidative phosphorylation (12 proteins, P<0.01), Parkinson's disease (12 proteins, P<0.01), Alzheimer's disease (11 proteins, P=0.01), and Huntington's disease (10 proteins, P=0.03). Protein-protein interactions identified between groups were also noted among the differential proteins (>1.2-fold or < 0.83-fold) (Figure 3(d)). String analyses show that the network of EA/CUMS has significantly more interactions than expected.

#### 3.3.3. Synaptic and Mitochondria Proteins Are Involved in EA Intervention

**Figure 4:**
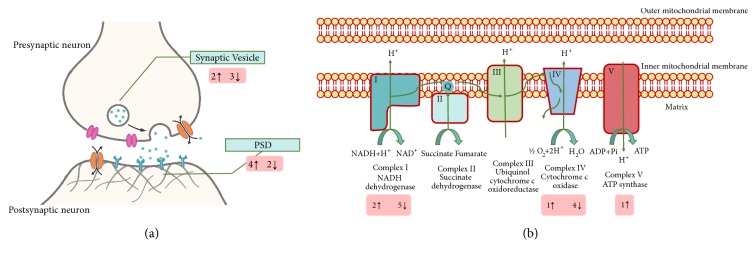
EA causes synaptic proteins and mitochondrial electron transport chain proteins change in depressed rats. (a) Synaptic proteins EA changed are mainly involved in PSD and synaptic vesicle. 5 synaptic vesicle proteins changed after EA, among which 2 were upregulated, while 3 were downregulated. 6 proteins concerning PSD were changed, and 4 of them were upregulated, while 2 were downregulated. (b) Among the differential expressed proteins distributed in mitochondria 2 of the proteins that consist of NADH dehydrogenase were upregulated while 5 were downregulated. In cytochrome c oxidase, 1 was upregulated, while 4 were downregulated. Only 1 protein that belongs to ATP synthase changed and it was upregulated. ↑: upregulated protein number. ↓: downregulated protein number. PSD: postsynaptic density.

Based on ultrastructure study findings, we focused on differential expressed protein in mitochondrion and synapse. Consisting with the transmission electron microscopy results, a total of 16 synaptic proteins were significantly changed by EA, among which 9 of them were upregulated and 7 were downregulated (with all proteins shown in Supplementary Data Tables 1 and 2). These proteins are mainly distributed in PSD and synaptic vesicle. 6 out of 16 proteins concerning PSD were changed, and 4 of them were upregulated, while 2 were downregulated; 5 synaptic vesicle proteins changed after EA, among which 2 were upregulated, while 3 were downregulated (Figure 4(a)).

On the other hand, 48 mitochondrial proteins changed after EA, of which 26 of them were upregulated and 22 were downregulated (with all proteins shown in Supplementary Data Tables 3 and 4). Interestingly, mitochondrial electron transport chain proteins alterations were rather distinct. 2 of the proteins consisting of NADH dehydrogenase were upregulated, while 5 were downregulated. In cytochrome c oxidase, 1 was upregulated, while 4 were downregulated. Only 1 protein that belongs to ATP synthase changed and it was upregulated (Figure 4(b)).

## 4. Discussion

Due to increasing concerns regarding the delay of antidepressant's effects, clinical researches posed a complimentary therapy combination strategy to overcome this, to combine SSRI with EA [7]. The combination therapy can bring the symptoms relief front to within 1 week [8, 9]. In this study, we showed that EA shortens the time lag of SSRI's effects. We also showed that EA has similar effects as SSRI in reducing anhedonia and anxiety as well as increasing bodyweight.

Notably, in bodyweight increasing, SSRIs precede EA and EA+SSRI. As a matter of fact, antidepressants, such as tricyclic antidepressants (TCAs) and SSRIs, are often related to weight gain in both acute and long-term treatments [38]. Paroxetine, SSRI used in this research, is associated with a greater risk of weight gain among antidepressants [39]. Thus, SSRIs may be a more favorable weight alternative than EA in patients who have significant weight loss. Still, EA+SSRI had less weight gain than SSRI. We therefore accumulate that EA may have more complicated effects on body weight in depression.

At excitatory synapses throughout the central nervous system, the molecular composition of the postsynaptic membrane is a key determinant of synaptic strength [40]. It is a huge protein complex associated with postsynaptic membranes of excitatory synapses composed of more than 1,000 proteins including receptors, scaffold proteins, signaling enzymes, and cytoskeletal proteins. These proteins are crucial for synaptic transmission and plasticity [41]. Increasing evidence shows that the depression and acting mechanism of EA are related to altered synaptic plasticity, especially in hippocampus [18, 19, 21–23]. Synaptic changes are determined by pre- and postsynaptic structures changes such as axonal bouton, dendritic spine, and PSD [42]. In our research, we have shown that EA and EA-SSRI combination reverse PSD loss caused by CUMS in hippocampal neuron. It is accompanied by PSD proteins change, confirming that hippocampal synaptic plasticity is involved in EA's effects. Although researches before find that EA works by altering serotonin or glutamate receptor proteins, we did not observe that these proteins changed after EA. On the other hand, postsynaptic scaffold proteins are mainly involved. We speculate that the difference came from intervention time, which of these researches were all above 3 weeks. In this study we only conducted EA intervention for 1 week. Regarding the major function of scaffold proteins which is anchoring and clustering receptors proteins [43], we expect receptor proteins to change following alteration of these scaffold proteins.

Mitochondria are highly dynamic organelles that divide, fuse, and move purposefully within axons and dendrites [44]. Mitochondrial electron transport generates the ATP which is essential for the excitability and survival of neurons and the protein phosphorylation reactions that mediate synaptic signaling and related long-term changes in neuronal structure and function [45]. In this research, we also find that EA reverses mitochondrial damage in hippocampal neuron during CUMS exposure. iTRAQ data reveal that the process is accompanied by electron transport chain changes, where NADH dehydrogenase changes significantly. This is an original study which shows that EA has protective effects on hippocampal mitochondrion. Regarding synaptic plasticity regulation which is one of the major functions of mitochondria in neurons, we assume mitochondria a possible important target of EA's synaptic plasticity mechanism.

As a matter of fact, mitochondria are essential organelles for synaptic plasticity. In synapse, mitochondria serve as energy/ATP supplier and calcium buffer organelles for long-term potentiation (LTP, effects show when synapse strengthens). On the other hand, the strengthening of synapse also promotes mitochondrial function and therefore increases ATP generation [46]. Moreover, altered mitochondrial membrane permeabilization releases cytochrome c (cyt-c). This activates caspases cascades (the intrinsic pathway), which then causes synaptic pruning (elimination of synapses) and induces long-term depression (LTD, effects shown when synapse weakens) [47]. Synaptic plasticity forms LTP and LTD are NMDA and AMPA receptors-dependent. AMPAR internalization and insertion in postsynaptic membrane and stabilization in PSD appear to be the primary cell biological mechanism underlying LTP and LTD. It is shown that AMPAR is caspase substrates. However, the exact mechanism of how caspase controls AMPAR trafficking is still unclear [48]. In our research, EA is shown to protect both mitochondria and synapse integrity in depressed hippocampus. Regarding the importance of mitochondria in synaptic plasticity, we assume that EA accelerates antidepressant effects by protecting synaptic plasticity in hippocampus via mitochondria.

Interestingly, although SSRI alone has less impact on increasing PSD than EA alone (Figure 2(a)), the effects of EA and SSRI on depressive symptoms remain similar in all behavioral tests, suggesting that the underlying mechanism of EA and SSRI may not be exactly the same. As a matter of fact, although SSRIs are generally believed to increase the level of serotonin in the synaptic cleft by limiting its reabsorption (reuptake) into the presynaptic cell, they have varying degrees of affinity for the other monoamine transporters [49, 50]. Therefore, their exact mechanism of action remains elusive. Recent researches suggest that SSRIs improve depression through anti-inflammatory [51–53]. In this study we also show that SSRI (Paroxetine) increases PSD and protects mitochondrion in hippocampal neuron, providing new insights for mechanism of SSRIs.

Despite the different synaptic or mitochondrial proteins, 30.29% (83) different proteins locate in cytoplasm, 29.93% (82) in membrane, part of which relate to synapse. We also find that clusters of protein network relate to protein synthesis (19 proteins, of which 5 are ribosome proteins, all upregulated), cell motility (19 proteins), and translation (14 proteins). To this day, we cannot find any evidence connecting these proteins with depression. The role of these proteins in EA anti-depression effects needs to be further discussed.

Thus, this research provides a strategy to overcome the effects delay of SSRI, to combine with EA. We also proposed EA as an alternative monotherapy therapy for depression. Moreover, it provides a valuable clinical reference concerning the mechanism of EA anti-depression; that is, EA changes hippocampal synaptic and mitochondrial proteomics profiles. In depressed hippocampus, synapse and mitochondrion may be the target organelles of EA. Our findings also show that altering hippocampal synaptic plasticity may be involved in the mechanism of SSRI.

Possible limitations of the study include the fact that the exact crucial protein that responds to EA's synaptic plasticity effects needs further discussion and verification. The connection between synapse and mitochondrion in EA intervention remains inconclusive, which requires further researches in the future. We hypothesized that EA reduces CUM-induced PSD loss through protecting mitochondria functions. Still, the exact mechanism remains to be further looked into, but the functions of electron transport chain located in mitochondrial inner membrane may be a promising direction. Besides, in the experiments, we used the whole hippocampus, which includes dentate gyrus (DG), CA1, CA2, CA3, and CA4. Regarding the fact that most of the results in this research are involved in synaptic plasticity, we would like to focus on CA1 and DG next time, for synaptic plasticity in CA1 is rather vulnerable in diseases and adult neurogenesis still exists in DG, which made DG a high synaptic plasticity subfield.

## Figures and Tables

**Table 1:** Differentially expressed proteins numbers between groups.

Compared groups	Upregulated protein number	Downregulated protein number
EA/CUMS	145	129

**Table 2:** KEGG analysis of differential expressed proteins between EA and CUMS rats.

ID	KEGG Pathway	Protein number	P value
ko04932	Nonalcoholic fatty liver disease (NAFLD)	12	<0.001
ko00190	Oxidative phosphorylation	12	<0.01
ko05012	Parkinson's disease	12	<0.01
ko05010	Alzheimer's disease	11	0.01
ko05016	Huntington's disease	10	0.03

## Data Availability

The datasets used and analyzed during the current study are available from the corresponding author on reasonable request.

## Supplementary Materials

Supplementary Materials Table 1: Upregulated synaptic proteins compared EA to CUMS. Table 2: Downregulated synaptic proteins compared EA to CUMS. Table 3: Upregulated mitochondrial proteins compared EA to CUMS. Table 4: Downregulated mitochondrial proteins compared EA to CUMS. Graphic abstract: EA: EA. Hipp: hippocampus. PSD: postsynaptic density.
