# Supplementary material for: Electroacupuncture Improves Antidepressant Effects in CUMS Rats by Protecting Hippocampal Synapse and Mitochondrion: An Ultrastructural and iTRAQ Proteomic Study
Source: Evid Based Complement Alternat Med. 2019 Apr 17;2019:3424698. doi: 10.1155/2019/3424698 (PMC6500624; doi:10.1155/2019/3424698)
Supplement: Supplementary Materials — Table 1: Upregulated synaptic proteins compared EA to CUMS. Table 2: Downregulated synaptic proteins compared EA to CUMS. Table 3: Upregulated mitochondrial proteins compared EA to CUMS. Table 4: Downregulated mitochondrial proteins compared EA to CUMS. Graphic abstract: EA: EA. Hipp: hippocampus. PSD: postsynaptic density. [file 3424698.f1.pdf]

Table 1. Up-regulated synaptic proteins compared EA to CUMS

Name	cellular component	biological process	molecular function
Heat shock cognate 71 kDa protein	dendrite;<br>synaptic vesicle;<br>**postsynaptic density;**<br>neuron projection	response to heat;<br>protein import into nucleus;<br>cerebellum development;<br>positive regulation of lysosomal membrane permeability;<br>response to activity;<br>response to odorant;<br>response to estradiol;<br>negative regulation of transcription, DNA-templated;<br>response to drug;<br>positive regulation of gene expression	ADP binding;<br>ATP binding;<br>receptor binding;<br>clathrin-uncoating ATPase activity;<br>prostaglandin binding;<br>RNA binding;<br>ATPase activity, coupled;<br>nucleotide binding;<br>unfolded protein binding;<br>transcription factor binding
Calponin-3	dendrite;<br>**postsynaptic density;**<br>neuronal cell body;<br>dendritic spine;<br>focal adhesion;<br>microtubule;<br>actin filament;<br>actin cytoskeleton	epithelial cell differentiation;<br>negative regulation of ATPase activity;<br>actomyosin structure organization	calmodulin binding;<br>microtubule binding;<br>actin binding;<br>protein binding, bridging
Abl interactor 1	**postsynaptic density**;<br>neuron projection	lamellipodium morphogenesis;<br>somitogenesis;<br>positive regulation of protein tyrosine kinase activity;<br>peptidyl-tyrosine phosphorylation;<br>cellular process;<br>megakaryocyte development	protein tyrosine kinase activator activity;<br>protein complex binding;<br>transcription factor binding;<br>protein binding
p55 protein	**postsynaptic density**		
COP9 signalosome complex subunit 4	**synaptic vesicle;**<br>synapse	protein deneddylation;<br>cullin deneddylation	NEDD8-specific protease activity;<br>protein binding
Phosphatidylethanolamine-binding protein 1	**synaptic vesicle;**<br>neuron projection;<br>axon terminus;<br>neuronal cell body;<br>myelin sheath	hippocampus development;<br>positive regulation of cAMP-mediated signaling;<br>negative regulation of peptidase activity;<br>spermatid development;<br>response to heat;<br>response to corticosterone;<br>response to organic cyclic compound;<br>positive regulation of mitotic nuclear division;<br>MAPK cascade;	ATP binding;<br>serine-type endopeptidase inhibitor activity;<br>receptor binding;<br>protein kinase binding;<br>mitogen-activated protein kinase binding;<br>nucleotide binding;<br>peptidase inhibitor activity;<br>lipid binding;<br>kinase binding

		response to organonitrogen compound	
Platelet glycoprotein Ib beta chain	**synaptic vesicle;**<br>axon terminus;<br>synapse	positive regulation of exocytosis;<br>regulation of exocytosis	syntaxin binding;<br>nucleotide binding;<br>GTP binding
Alpha-synuclein	**synaptic vesicle;**<br>axon terminus;<br>cytoskeleton	regulation of dopamine secretion;<br>dopamine biosynthetic process;<br>response to interleukin-1;<br>positive regulation of peptidyl-serine phosphorylation;<br>activation of cysteine-type endopeptidase activity involved in apoptotic process;<br>synaptic vesicle transport;<br>long-term synaptic potentiation;<br>regulation of neuronal synaptic plasticity;<br>negative regulation of platelet-derived growth factor receptor signaling pathway;<br>response to iron(II) ion	dynein binding;<br>beta-tubulin binding;<br>identical protein binding;<br>magnesium ion binding;<br>cysteine-type endopeptidase inhibitor activity involved in apoptotic process;<br>calcium ion binding;<br>phospholipase binding;<br>arachidonic acid binding;<br>alpha-tubulin binding;<br>copper ion binding
Gamma-enolase	synaptic membrane;<br>neuron projection	glycolytic process;<br>gluconeogenesis;<br>response to organic cyclic compound;<br>response to estradiol;<br>response to drug	magnesium ion binding;<br>protein heterodimerization activity;<br>lyase activity;<br>protein homodimerization activity;<br>phosphopyruvate hydratase activity;<br>metal ion binding
NADH dehydrogenase (Ubiquinone) Fe-S protein 7	synaptic membrane;<br>neuron projection;<br>neuronal cell body	mitochondrial respiratory chain complex I assembly;<br>oxidation-reduction process	quinone binding;<br>iron-sulfur cluster binding;<br>NADH dehydrogenase (ubiquinone) activity;<br>NADH dehydrogenase activity;<br>oxidoreductase activity;<br>4 iron, 4 sulfur cluster binding;<br>protease binding;<br>metal ion binding

Table 2.. Down-regulated synaptic proteins compared EA to CUMS

Name	cellular component	biological process	molecular function
Potassium voltage-gated channel subfamily D member 2	dendrite;<br>**postsynaptic density;**<br>neuron projection;<br>voltage-gated potassium channel complex;<br>perikaryon;	protein heterooligomerization;<br>transport;<br>protein homooligomerization;<br>cellular response to hypoxia;<br>transmembrane transport;<br>ion transport;<br>potassium ion transport;	ion channel activity;<br>A-type (transient outward) potassium channel activity;<br>potassium channel activity;<br>protein heterodimerization activity;<br>voltage-gated potassium channel activity;

	cell junction;<br>neuronal cell body membrane	action potential;<br>cardiac muscle cell action potential;<br>potassium ion transmembrane transport	delayed rectifier potassium channel activity;<br>protein binding;<br>voltage-gated ion channel activity;<br>metal ion binding
Protein Neto1	**postsynaptic density;**<br>synapse;<br>excitatory synapse;<br>**kainate selective glutamate receptor complex**	regulation of neuronal synaptic plasticity;<br>regulation of long-term neuronal synaptic plasticity;<br>visual learning;<br>receptor localization to synapse;<br>positive regulation of excitatory postsynaptic potential;<br>memory	ionotropic glutamate receptor binding
Ras-related protein Rab-8B	**synaptic vesicle**	protein transport;<br>adherens junction organization;<br>protein secretion;<br>vesicle docking involved in exocytosis;<br>positive regulation of cell projection organization;<br>small GTPase mediated signal transduction;<br>transport;<br>cellular response to insulin stimulus;<br>regulation of exocytosis;<br>synaptic vesicle exocytosis	GDP binding;<br>GTPase activity;<br>receptor binding;<br>nucleotide binding;<br>protein binding;<br>TPR domain binding;<br>GTP binding
Protein Sypl1	**synaptic vesicle**	transport	transporter activity
Protein Syngr3	**synaptic vesicle;**<br>synaptic vesicle membrane;<br>synapse	substantia nigra development;<br>positive regulation of transporter activity	SH2 domain binding;<br>protein N-terminus binding
Brefeldin A-inhibited guanine nucleotide-exchange protein 2	dendrite;<br>symmetric synapse;<br>cytoskeleton	positive regulation of tumor necrosis factor production;<br>protein transport;<br>receptor recycling;<br>transport;<br>positive regulation of GTPase activity;<br>regulation of ARF protein signal transduction;<br>exocytosis;<br>endomembrane system organization;<br>Golgi to plasma membrane transport;<br>intracellular signal transduction	guanyl-nucleotide exchange factor activity;<br>GABA receptor binding;<br>myosin binding;<br>protein kinase A regulatory subunit binding;<br>ARF guanyl-nucleotide exchange factor activity;<br>protein binding
Sodium/hydrogen exchanger	dendrite;<br>axon terminus;<br>synapse	neuron projection morphogenesis;<br>sodium ion transport;<br>transport;<br>regulation of pH;	solute:proton antiporter activity;<br>sodium:proton antiporter activity;<br>antiporter activity

		regulation of neurotrophin TRK receptor signaling pathway;<br>synapse organization;<br>transmembrane transport;<br>dendritic spine development;<br>ion transport;<br>axon extension	

Table 3. Up-regulated mitochondrial proteins compared EA to CUMS

Name	cellular component	biological process	molecular function
NADH dehydrogenase (Ubiquinone) Fe-S protein 8 (Predicted), isoform CRA a	mitochondrial respiratory chain **complex I;**	mitochondrial respiratory chain complex I;	NADH dehydrogenase (Ubiquinone) Fe-S protein 8 (Predicted), isoform CRA a
NADH dehydrogenase (Ubiquinone) Fe-S protein 7	mitochondrial respiratory chain **complex I;**<br>synaptic membrane;<br>neuron projection;<br>mitochondrial inner membrane;<br>mitochondrion;<br>neuronal cell body	mitochondrial respiratory chain complex I assembly;<br>oxidation-reduction process	quinone binding;<br>iron-sulfur cluster binding;<br>NADH dehydrogenase (ubiquinone) activity;<br>NADH dehydrogenase activity;<br>oxidoreductase activity;<br>4 iron, 4 sulfur cluster binding;<br>protease binding;<br>metal ion binding
ATP synthase subunit beta	mitochondrial inner membrane;<br>mitochondrial nucleoid;<br>mitochondrion;<br>plasma membrane;<br>proton-transporting **ATP synthase complex, catalytic core F(1);**<br>proton-transporting two-sector ATPase complex, catalytic domain;<br>myelin sheath;<br>mitochondrial proton-transporting ATP synthase complex	osteoblast differentiation;<br>angiogenesis;<br>ATP biosynthetic process;<br>transport;<br>ATP synthesis coupled proton transport;<br>regulation of intracellular pH;<br>ion transport;<br>negative regulation of cell adhesion involved in substrate-bound cell migration;<br>lipid metabolic process;<br>ATP hydrolysis coupled proton transport	ATP binding;<br>proton-transporting ATPase activity, rotational mechanism;<br>proton-transporting ATP synthase activity, rotational mechanism;<br>nucleotide binding;<br>MHC class I protein binding;<br>ATPase activity;<br>hydrolase activity, acting on acid anhydrides, catalyzing transmembrane movement of substances
Cytochrome c oxidase subunit 6B1	mitochondrion	hydrogen ion transmembrane transport	cytochrome-c oxidase activity
Pyruvate dehydrogenase E1 component subunit	mitochondrion;<br>mitochondrial matrix;<br>pyruvate	glucose metabolic process;<br>metabolic process;<br>carbohydrate metabolic process;	catalytic activity;<br>pyruvate dehydrogenase (acetyl-transferring) activity;

beta, mitochondrial	dehydrogenase complex	tricarboxylic acid cycle;<br>oxidation-reduction process;<br>acetyl-CoA biosynthetic process from pyruvate	oxidoreductase activity;<br>pyruvate dehydrogenase activity
Dihydrolipoamide S-succinyltransferase (E2 component of 2-oxo-glutarate complex), isoform CRA a	mitochondrion;<br>oxoglutarate dehydrogenase complex;<br>membrane;<br>myelin sheath	metabolic process;<br>tricarboxylic acid cycle;<br>2-oxoglutarate metabolic process;<br>NADH metabolic process	chaperone binding;<br>transferase activity;<br>heat shock protein binding;<br>transferase activity, transferring acyl groups;<br>dihydrolipoyllysine-residue succinyltransferase activity
GM2 ganglioside activator	mitochondrion;<br>hydrogen:potassium-exchanging ATPase complex	neurological system process;<br>ganglioside catabolic process;<br>lipid transport;<br>ganglioside metabolic process;<br>membrane fusion;<br>oligosaccharide catabolic process;<br>positive regulation of hydrolase activity;<br>neuromuscular process controlling balance;<br>lipid storage;<br>learning or memory	lipid transporter activity;<br>beta-N-acetylhexosaminidase activity;<br>beta-N-acetylgalactosaminidase activity;<br>phospholipase activator activity;<br>enzyme activator activity
Carboxypeptidase	mitochondrion	proteolysis	serine-type carboxypeptidase activity
Thioredoxin	dendrite;<br>mitochondrion;<br>neuronal cell body;<br>axon	cellular response to glucose stimulus;<br>positive regulation of DNA binding;<br>response to activity;<br>cell redox homeostasis;<br>regulation of protein import into nucleus, translocation;<br>response to drug;<br>cellular response to hyperoxia;<br>response to selenium ion;<br>response to axon injury;<br>sulfate assimilation	thioredoxin-disulfide reductase activity;<br>oxidoreductase activity, acting on a sulfur group of donors, disulfide as acceptor;<br>protein disulfide oxidoreductase activity;<br>enzyme binding
Phosphatidylethanolamine-binding protein 1	synaptic vesicle;<br>neuron projection;<br>mitochondrion;<br>axon terminus;<br>mitochondrial outer membrane;<br>membrane;<br>neuronal cell body;<br>myelin sheath	hippocampus development;<br>positive regulation of cAMP-mediated signaling;<br>negative regulation of peptidase activity;<br>spermatid development;<br>response to heat;<br>response to corticosterone;<br>response to organic cyclic compound;<br>positive regulation of mitotic nuclear	ATP binding;<br>serine-type endopeptidase inhibitor activity;<br>receptor binding;<br>protein kinase binding;<br>mitogen-activated protein kinase binding;<br>nucleotide binding;<br>peptidase inhibitor activity;<br>lipid binding;<br>kinase binding

		division;<br>MAPK cascade;<br>response to organonitrogen compound	
Malate dehydrogenase, mitochondrial	mitochondrial inner membrane;<br>mitochondrion;<br>plasma membrane;<br>myelin sheath;<br>mitochondrial matrix	oxaloacetate metabolic process;<br>carbohydrate metabolic process;<br>tricarboxylic acid cycle;<br>oxidation-reduction process;<br>NADH metabolic process;<br>internal protein amino acid acetylation;<br>malate metabolic process;<br>carboxylic acid metabolic process	catalytic activity;<br>poly(A) RNA binding;<br>L-malate dehydrogenase activity;<br>malate dehydrogenase (NADP+) activity;<br>malate dehydrogenase activity;<br>oxidoreductase activity;<br>protein self-association;<br>protein binding;<br>oxidoreductase activity, acting on the CH-OH group of donors, NAD or NADP as acceptor
Dihydrolipoyllysine-residue acetyltransferase component of pyruvate dehydrogenase complex, mitochondrial	mitochondrion;<br>myelin sheath;<br>mitochondrial matrix;<br>pyruvate dehydrogenase complex	glucose metabolic process;<br>metabolic process;<br>carbohydrate metabolic process;<br>sleep;<br>tricarboxylic acid cycle;<br>pyruvate metabolic process;<br>acetyl-CoA biosynthetic process from pyruvate	dihydrolipoyllysine-residue acetyltransferase activity;<br>transferase activity;<br>transferase activity, transferring acyl groups;<br>nucleotide binding
Protein FAM136A	mitochondrion		
Long-chain fatty acid transport protein 1	mitochondrial inner membrane;<br>mitochondrion;<br>plasma membrane;<br>integral component of membrane	metabolic process;<br>lipid transport;<br>transport;<br>long-chain fatty acid metabolic process;<br>lipid metabolic process;<br>fatty acid transport;<br>fatty acid metabolic process	catalytic activity;<br>fatty acid transporter activity;<br>nucleotide binding;<br>ligase activity;<br>protein binding;<br>long-chain fatty acid-CoA ligase activity;<br>very long-chain fatty acid-CoA ligase activity
Protein Dnajc19	mitochondrial inner membrane;<br>mitochondrion	visual perception;<br>protein import into mitochondrial matrix;<br>positive regulation of ATPase activity;<br>genitalia development	protein transporter activity;<br>ATPase activator activity
ADP-ribosylhydrolase like 2	mitochondrion	metabolic process;<br>cellular response to superoxide	hydrolase activity
Hydroxyacyl glutathione hydrolase	mitochondrion	carbohydrate metabolic process;<br>spermatogenesis;<br>glutathione metabolic process;<br>methylglyoxal catabolic process to D-lactate via S-lactoyl-glutathione	hydroxyacylglutathione hydrolase activity;<br>hydrolase activity
Endophilin-B1	mitochondrion;<br>mitochondrial outer	apoptotic process	lipid binding

	membrane;<br>membrane;<br>autophagosome		
Alpha-synuclein	synaptic vesicle;<br>mitochondrion;<br>axon terminus;<br>cytoskeleton	regulation of dopamine secretion;<br>dopamine biosynthetic process;<br>response to interleukin-1;<br>positive regulation of peptidyl-serine phosphorylation;<br>activation of cysteine-type endopeptidase activity involved in apoptotic process;<br>synaptic vesicle transport;<br>long-term synaptic potentiation;<br>regulation of neuronal synaptic plasticity;<br>negative regulation of platelet-derived growth factor receptor signaling pathway;<br>response to iron(II) ion	dynein binding;<br>beta-tubulin binding;<br>identical protein binding;<br>magnesium ion binding;<br>cysteine-type endopeptidase inhibitor activity involved in apoptotic process;<br>calcium ion binding;<br>phospholipase binding;<br>arachidonic acid binding;<br>alpha-tubulin binding;<br>copper ion binding
Mitochondrial fission regulator 1-like	mitochondrion	mitochondrial fission;	Mitochondrial fission regulator 1-like
1-phosphatidylinositol 4,5-bisphosphate phosphodiesterase delta-1	mitochondrial membrane	response to prostaglandin F;<br>regulation of phospholipase C activity;<br>response to organonitrogen compound;<br>response to aluminum ion;<br>positive regulation of cytosolic calcium ion concentration involved in phospholipase C-activating G-protein coupled signaling pathway;<br>response to hyperoxia;<br>lipid catabolic process;<br>G-protein coupled receptor signaling pathway;<br>signal transduction;<br>positive regulation of inositol trisphosphate biosynthetic process	phosphoric diester hydrolase activity;<br>calcium ion binding;<br>signal transducer activity;<br>phosphatidylinositol phospholipase C activity;<br>phospholipid binding;<br>hydrolase activity;<br>protein binding;<br>phosphatidylinositol-4,5-bisphosphate binding;<br>metal ion binding
Mitochondrial ribosomal protein L4 (Predicted), isoform CRA b	mitochondrion	translation	structural constituent of ribosome;<br>poly(A) RNA binding;<br>RNA binding
Peptidylprolyl isomerase	neuron projection;<br>mitochondrion	positive regulation of ubiquitin-protein transferase activity;<br>metabolic process;<br>negative regulation of cell motility;<br>positive regulation of neuron apoptotic process;<br>negative regulation of neuron	mitogen-activated protein kinase kinase binding;<br>phosphoserine binding;<br>phosphothreonine binding;<br>isomerase activity;<br>protein binding;<br>GTPase activating protein binding;

		apoptotic process;<br>negative regulation of ERK1 and ERK2 cascade;<br>positive regulation of cell growth involved in cardiac muscle cell development;<br>positive regulation of GTPase activity;<br>protein peptidyl-prolyl isomerization;<br>regulation of cytokinesis	peptidyl-prolyl cis-trans isomerase activity
Protein Slirp	mitochondrion;<br>intracellular	spermatid development;<br>mitochondrion morphogenesis;<br>negative regulation of mitochondrial RNA catabolic process;<br>sperm motility;<br>single fertilization	poly(A) RNA binding;<br>nucleotide binding;<br>nucleic acid binding
Mitochondrial import inner membrane translocase subunit Tim13	mitochondrial inner membrane;<br>mitochondrion	protein transport;<br>transport	metal ion binding

Table 4. Down-regulated mitochondrial proteins compared EA to CUMS

Name	cellular component	biological process	molecular function
NADH-ubiquinone oxidoreductase chain 1	mitochondrial inner membrane;<br>mitochondrion	oxidation-reduction process	NADH dehydrogenase (ubiquinone) activity;<br>oxidoreductase activity
NADH-ubiquinone oxidoreductase chain 4	mitochondrion	oxidation-reduction process;<br>mitochondrial electron transport, NADH to ubiquinone;<br>ATP synthesis coupled electron transport	NADH dehydrogenase (ubiquinone) activity;<br>oxidoreductase activity
NADH-ubiquinone oxidoreductase chain 5	mitochondrial inner membrane;<br>mitochondrion;<br>respiratory chain	oxidation-reduction process;<br>ATP synthesis coupled electron transport	NADH dehydrogenase (ubiquinone) activity;<br>oxidoreductase activity
NADH dehydrogenase [ubiquinone] flavoprotein 3, mitochondrial	mitochondrial respiratory chain complex I;<br>mitochondrial inner membrane;<br>mitochondrion;<br>respiratory chain	oxidation-reduction process	NADH dehydrogenase [ubiquinone] flavoprotein 3, mitochondrial OS=Rattus norvegicus GN=Ndufv3 PE=3 SV=1 - [NDUV3_RAT]
NADH dehydrogenase [ubiquinone] 1 alpha subcomplex subunit 9,	mitochondrial respiratory chain complex I;	response to glucose;<br>ubiquinone-6 biosynthetic process;<br>oxidation-reduction process	NADH dehydrogenase activity

mitochondrial	mitochondrion;<br>respiratory chain;<br>mitochondrial matrix		
Cytochrome c oxidase subunit 7B, mitochondrial	mitochondrial inner membrane;<br>mitochondrion;<br>integral component of membrane;<br>respiratory chain complex IV;<br>mitochondrial respiratory chain	central nervous system development;<br>hydrogen ion transmembrane transport	cytochrome-c oxidase activity
Cytochrome c oxidase subunit 7C, mitochondrial	mitochondrial inner membrane;<br>mitochondrion;<br>integral component of membrane	hydrogen ion transmembrane transport	cytochrome-c oxidase activity
Cytochrome c oxidase subunit 6A1, mitochondrial	mitochondrial inner membrane;<br>mitochondrion;<br>mitochondrial respiratory chain complex IV	hydrogen ion transmembrane transport	cytochrome-c oxidase activity
Cytochrome c oxidase subunit 7A2, mitochondrial	mitochondrial inner membrane;<br>mitochondrion;<br>membrane;<br>mitochondrial respiratory chain	hydrogen ion transmembrane transport	cytochrome-c oxidase activity;<br>electron carrier activity
Phospholipid scramblase 3	mitochondrion;<br>mitochondrial membrane	glucose homeostasis;<br>apoptotic process;<br>phospholipid scrambling;<br>cellular response to lipopolysaccharide;<br>cholesterol homeostasis	phospholipid scramblase activity;<br>SH3 domain binding;<br>calcium-dependent protein binding
Nucleoside diphosphate kinase	mitochondrion	nucleoside diphosphate phosphorylation;<br>UTP biosynthetic process;<br>phosphorylation;<br>GTP biosynthetic process;<br>CTP biosynthetic process	ATP binding;<br>nucleoside diphosphate kinase activity;<br>kinase activity;<br>transferase activity;<br>nucleotide binding
Protein Tomm7	mitochondrion;<br>integral component of mitochondrial outer membrane;<br>mitochondrial outer membrane translocase complex	positive regulation of protein targeting to mitochondrion;<br>protein import into mitochondrial matrix;<br>mitophagy in response to mitochondrial depolarization;<br>protein import into mitochondrial outer membrane;	protein channel activity

		regulation of protein stability	
Mitochondrial pyruvate carrier 2	mitochondrial inner membrane;<br>mitochondrion	transport;<br>pyruvate transmembrane transport;<br>pyruvate metabolic process;<br>mitochondrial pyruvate transport	pyruvate transmembrane transporter activity
Ras-related protein Rab-8B	synaptic vesicle;<br>mitochondrion	protein transport;<br>adherens junction organization;<br>protein secretion;<br>vesicle docking involved in exocytosis;<br>positive regulation of cell projection organization;<br>small GTPase mediated signal transduction;<br>transport;<br>cellular response to insulin stimulus;<br>regulation of exocytosis;<br>synaptic vesicle exocytosis	GDP binding;<br>GTPase activity;<br>receptor binding;<br>nucleotide binding;<br>protein binding;<br>TPR domain binding;<br>GTP binding
Solute carrier family 25 member 51	mitochondrial inner membrane	mitochondrial inner membrane;	Solute carrier family 25 member 51
Serine/threonine-protein kinase A-Raf	mitochondrion;<br>cytosol	MAPK cascade;<br>phosphorylation;<br>regulation of proteasomal ubiquitin-dependent protein catabolic process;<br>activation of MAPKK activity;<br>signal transduction;<br>regulation of TOR signaling;<br>protein phosphorylation;<br>intracellular signal transduction	protein kinase activity;<br>ATP binding;<br>MAP kinase kinase kinase activity;<br>protein serine/threonine kinase activity;<br>kinase activity;<br>transferase activity;<br>nucleotide binding;<br>receptor signaling protein activity;<br>metal ion binding
Myelin-associated oligodendrocyte basic protein	mitochondrion;<br>myelin sheath	nervous system development	structural constituent of myelin sheath
Protein Nars	mitochondrion	asparaginyl-tRNA aminoacylation;<br>tRNA aminoacylation for protein translation;<br>translation	aminoacyl-tRNA ligase activity;<br>ATP binding;<br>asparagine-tRNA ligase activity;<br>nucleotide binding;<br>nucleic acid binding;<br>ligase activity
Mitochondrial fission 1 protein	mitochondrion;<br>integral component of mitochondrial outer membrane;<br>peroxisome;<br>mitochondrial outer membrane	regulation of mitochondrion organization;<br>positive regulation of protein targeting to membrane;<br>peroxisome fission;<br>positive regulation of mitochondrial calcium ion concentration;<br>positive regulation of cysteine-type endopeptidase activity involved in apoptotic process;	receptor binding

		positive regulation of intrinsic apoptotic signaling pathway; protein homooligomerization; mitochondrion morphogenesis; negative regulation of endoplasmic reticulum calcium ion concentration; protein targeting to mitochondrion	
Growth hormone-inducible transmembrane protein	mitochondrial inner membrane; mitochondrion	apoptotic process	
Sodium/hydrogen exchanger	dendrite; mitochondrion; axon terminus; synapse	neuron projection morphogenesis; sodium ion transport; transport; regulation of pH; regulation of neurotrophin TRK receptor signaling pathway; synapse organization; transmembrane transport; dendritic spine development; ion transport; axon extension	solute:proton antiporter activity; sodium:proton antiporter activity; antiporter activity

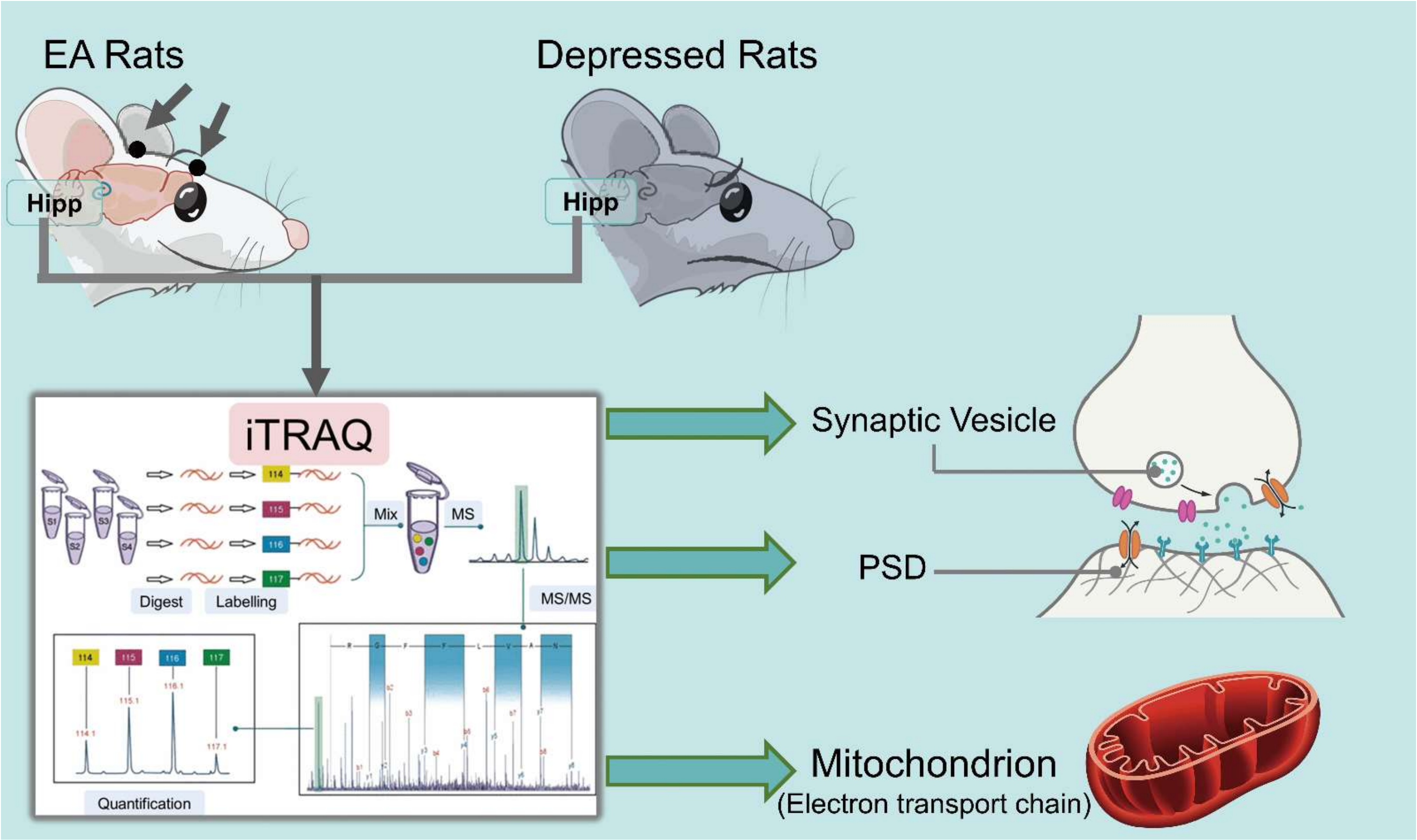

Graphic abstract

EA: EA. Hipp, hippocampus. PSD: postsynaptic density.
